# Quality of life following surgical pudendal nerve decompression

**DOI:** 10.1016/j.bas.2026.106051

**Published:** 2026-04-22

**Authors:** Patrick Dömer, Jennet Rahmanova, Thomas Kretschmer, Christian Heinen

**Affiliations:** a Department of Neurosurgery, Evangelisches Krankenhaus, University of Oldenburg, Germany; b Department of Neurosurgery and Neurorestoration, Neurosurgical Intensive Care, Klinikum Klagenfurt, Austria; c Department of Nerve-, Spine and Neurosurgery, Christliches Krankenhaus Quakenbrück, Germany

**Keywords:** Pudendal neuralgia, Quality of life, Surgical nerve decompression, Sacrotuberous and sacrospinal ligament

## Abstract

• PN rare and underdiagnosed.
• Retrospecticve study quality of life in PN.
• Special questionnaire to assess outcome after surgey.

## 1 Introduction

Pudendal neuralgia (PN) is a debilitating and underdiagnosed chronic pelvic pain syndrome. Its complex etiology, the various clinical manifestations and the absence of universally accepted diagnostic criteria is challenging for all persons involved. Not by chance, PN is one of the most controversially discussed compression neuropathy. As a result, individuals suffering from PN often suffer prolonged periods of physical and emotional distress before yielding accurate diagnosis and effective treatment.

The main feature of this condition is a neuralgic pain in the supply area of the pudendal nerve, which manifests in the region genitalia, perineum, rectum, or lower urinary tract (Roche et al., 2005). PN arises from the entrapment, irritation, or injury of the pudendal nerve, leading to a wide array of symptoms such as sharp or burning pain, numbness, and altered sensation in the affected areas due to the pivotal role of the pudendal nerve in sensory and motor functions in the pelvic region. The intensity and chronicity of these symptoms can significantly impact the quality of life, impairing daily activities, work, and interpersonal relationships.

The prevalence of PN remains unknown, although it is estimated by 1 in 100.000 (Hibner et al., 2010). However, the incidence of pudendal nerve injuries is expected to raise because of the increase of non-physiological sitting loads (Giulioni et al., 2024). Additionally, due to its anatomical location, the pudendal nerve is susceptible to damage during childbirth and surgical procedures (PETERS et al., 2014). Diagnosis of pudendal neuralgia primarily relies on clinical symptoms. Based on their huge experience, Labat et al. (2007) introduced the Nantes Criteria for the diagnosis of PN (Labat et al., 2007). These include essential features such as sensory abnormalities, exacerbation of symptoms with sitting along, positive effect of targeted pudendal nerve blockade in addition to supplementary criteria, exclusion criteria, and associated signs (for further details see Labat et al., 2007).

Pudendal nerve block is highly important in both diagnostic and therapeutic ways and are fundamental in the diagnostic of PN. They also are part of the conservative treatment with CT/X-ray guided or transvaginal/-rectal infiltrations treatment using corticosteroids and long-acting local anesthetics (Mamlouk et al., 2014). However, first line treatment is pain medication and physiotherapy (Levesque et al., 2022).

After failure of conservative therapy, surgical nerve decompression is an alternative for patients who have experienced temporary or partial improvement following pudendal nerve block.

The pudendal nerve can be compressed at several anatomical sites along its course - between the sacrospinous and sacrotuberous ligaments as it wraps around the ischial spine, the Alcock's canal and more distally e.g. due to varicosis.

Surgically, the nerve can be addressed via a transgluteal or a paraperineal surgical access (Tricard et al., 2018; Mauillon et al., 1999; Robert et al. 1998, 2004; Bautrant et al., 2003). The paraperineal approach allows for decompression in the Alcock's canal and the distal segments, but the possible proximal compression sites in the sacrotuberal region are not reachable. In contrast, the transgluteal approach provides a favorable view of the sacrotuberous and sacrospinal ligament (Roche et al., 2005) but does not facilitate the exposure of the distal Alcock canal. Moreover, the pudendal nerve can be operated on using the laparoscopic technique.

As mentioned, PN is significantly impairing quality of life (QoL). However, little is known and published on the mid- and long-term effects of patient's QoL.

Therefore, our group developed questionnaire-based assessment focused on two main criteria a) pain intensity and b) quality of life (QoL) of patients who underwent surgical decompression of pudendal nerves.

## 2 Methods

### 2.1 Study design

This retrospective and consecutive observational study received ethical approval from the Ethics Committee of Faculty IV, Medicine and Health Sciences, at Carl von Ossietzky University in Oldenburg, with the registration number 2020-089. The inclusion criteria involved patients undergoing surgical decompression of the pudendal nerve at the University Clinic for Neurosurgery, Evangelisches Krankenhaus Oldenburg, between 2015 and 2020.

### 2.2 Patient management

All patients with suspected PN were seen by an interdisciplinary team including neurosurgeons, gynecologists, urologists, neurologists, psychologists and gastroenterologists. All patients received pelvic MRI as routine imaging. Before surgery was indicated, one mandatory CT-guided pudendal nerve blockades were applied, in women that could be replaced by a transvaginal pudendal nerve blockade by our gynecologist (see Table 1 for our treatment algorithm).

**Table 1:**
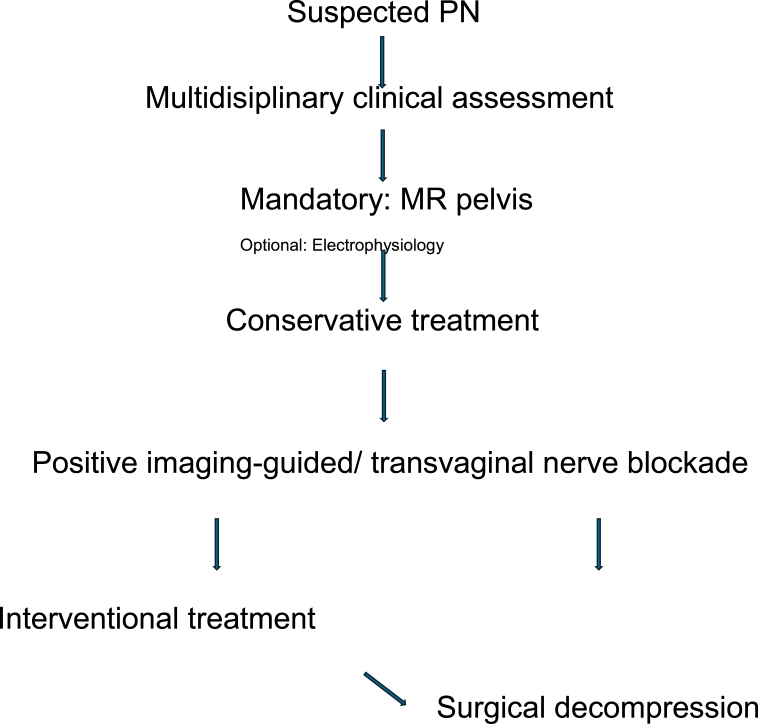
Flow chart of our treatment algorithm.

Pudendal nerve decompression was performed using the transgluteal muscle-sparing microsurgical approach as described by Robert et al. (2005). The paraperineal approach was applied either in revision surgery or in patients with a history of distal nerve trauma.

### 2.3 QoL assessment

The questionnaire was modified from the already validated German Pain Questionnaire (Pfingsten et al., 2006) which is tailored to chronic pain patients. Additionally, specific questions addressing the symptoms of PN patients were adapted (German and English Version available in the Supplement) following consultation with the German Pain Society e.V.

The patients were asked about changes in pain characteristics, intensity, and situations, as well as the impact of symptoms on daily activities, leisure pursuits, and overall well-being. The questions pertain to the condition before and after pudendal nerve decompression. Pain intensities were queried based on the visual analogue scale (VAS) ranging from 0 (no pain) to 10 (greatest imaginable pain).

The well-being was assessed using questions of the validated Marburg habitual well-being questionnaire (Basler, 1999). This questionnaire consisted of seven statements. On the six-point numerical rating scale (0 = not at all true and 5 = completely true), patients indicated the extent to which the statements applied to their well-being pre- and post-operatively. The estimated time required for completing the total questionnaire is approximately 15 min.

## 3 Results

### 3.1 Patient collective

From 2015 to 2020, a total of n = 61 patients presented with suspected PN in our outpatient department for consultation. After thorough examination and undergoing the interdisciplinary assessment, decompression of the pudendal nerve was performed on a total of n = 29 patients with pudendal neuralgia. For follow-up, n = 2 declined postoperative assessment, n = 1could not be contacted, and n = 1 patient passed away during follow up. The cause of death of this patient was not related to pudendal neuralgia and the surgery. Thus, n = 25 patients (n = 15 female, n = 10 male) with a total of n = 31 surgeries for decompression of the pudendal nerve were included. Mean patient age was 53 years (range: 28-70 years). All n = 25 participants exhibited a minimum of one year of pain symptoms and clinical manifestations of pudendal neuralgia, such as an exacerbation of symptoms in a seated position. Additionally, all patients (except one individual) responded positively to pudendal nerve blockade (after long discussions, we opted for surgery due to the clinical appearance). Pain intensity and duration prior to surgery were documented. Mean follow up period was 2.3 years (range 1.0 -5.3 years). N = 13 (n = 6 right side, n = 7 left side) and n = 12 bilateral decompressions of the pudendal nerve were performed. Among the bilaterally operated patients, n = 2 individuals underwent a total of n = 3 unilateral decompressions, while n = 2 other patients underwent n = 2 unilateral decompressions each. No complications during or after the operation was observed for any of the patients. Except for one patient who explicitly refused (see above), all patients had undergone at least one preoperative pudendal block. In women, the block was primarily transvaginal, while in men, it was CT-guided. All patients responded positively to the diagnostic pudendal block, experiencing a reduction in pain symptoms of at least 50%.

Since most of the patients came from all over, we cannot provide data on the conservatively treated patients as they were not followed up in our institution.

### 3.2 Preoperative pain symptoms

The patients reported pain in the perineal, genital, and anorectal regions, along with sensations of anal foreign body, as well as vaginal or scrotal discomfort. N = 5 patients (20%) additionally exhibited sensory deficits, such as numbness and tingling dysesthesia in the nerve's innervation area. Another n = 6 (24%) experienced motor disturbances, including sphincter spasms, bladder emptying disorders, persistent genital arousal, increased urinary urgency, and in the case of men, erectile dysfunction.

### 3.3 Association of trauma and PN

In n = 19 patients (76%), there was no direct pelvic trauma or similar cause for the pain, indicating no immediate trigger for PN. N = 6 patients (24%) reported symptom onset following traumatic events or surgical procedures. In all these cases, MR showed scarring in the pudendal area, whereas the MR of the other patients was just excluding other pathologies. Symptoms in n = 1 affected individual emerged after a fall from a ladder, while in n = 5 other patients, they arose after pelvic surgeries, including prostatectomy, bladder prolapse surgery, varicocele surgery, and pelvic radio- or thermotherapy.

### 3.4 Postoperative QoL assessment

**Fig. 1:**
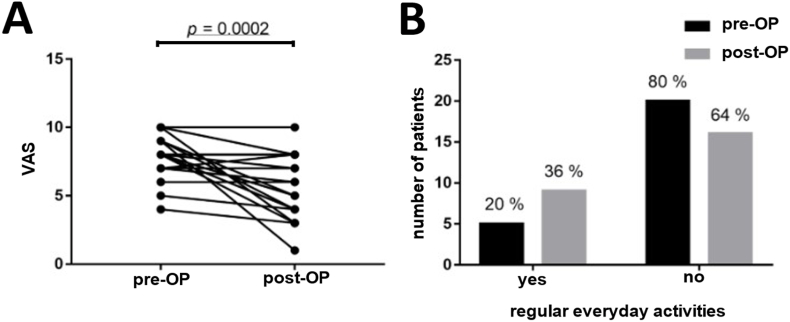
**A)** Pain intensity on the visual analogue scale. A value of 0 corresponded to complete freedom from pain, while a value of 10 represented the greatest imaginable pain.

Postoperatively, n = 15 (60%) of the patients experienced a decrease in pain intensity following the procedure (Fig. 1A). N = 1 patient achieved nearly complete pain relief, reducing symptoms from VAS 9 to 1. In n = 8 other patients there was a significant average reduction in pain intensity from VAS 8.5 to 4.1. Among n = 6 patients, an improvement in pain intensity was noted, decreasing on average from VAS 7.3 to 6. Pain intensity remained unchanged postoperatively for n = 9 individuals, and n = 1 person experienced an increase from VAS 7 to 8.

Out of the n = 25 patients, n = 18 reported having preoperative pain attacks (with and without persistent pain), and postoperatively, still n = 17 patients experienced them. N = 1 person had postoperative persistent pain without pain attacks. A positive change, indicating a reduction in the frequency of pain attacks, was observed in a total of n = 6 individuals.

Prior to PN surgery, n = 20 patients (80%) reported being unable to engage in everyday activities regularly due to their symptoms (Fig. 1B). Postoperatively, n = 15 patients (60%) were still unable to engage regularly in everyday activities. The situation worsened for n = 1 individual (4%), leading to an inability to pursue regular daily activities. N = 9 patients (36%) were able to perform daily activities. This improvement allowed n = 5 patients to resume their daily routines postoperatively.

Pharmacological therapy was reduced in n = 10 patients postoperatively (40%), whereas n = 7 patients (28%) continued their medications unchanged. Medication therapy was increased for n = 8 patients (32%).

Postoperatively, a significant improvement (p = 0.0001) in overall well-being was observed. N = 17 individuals (68%) showed an increased their overall well-being after the surgery. N = 4 patients (16%) reported no change, while another n = 4 (16%) suffered from deterioration. Within the cohort, significant shifts in agreement (0 = not at all true, 5 = completely true) were noted for the following fields: enjoyment of life (pre-OP: 1.24, post-OP: 2.08, p = 0.005), feeling comfortable (pre-OP: 1.4, post-OP: 2.08, p = 0.019) satisfaction with the work performance (pre-OP: 1.88, post-OP: 2.64, p = 0.004), satisfaction with the physical condition (pre-OP: 0.88, post-OP:1.76, p = 0.005), and the experience of true joy (pre-OP: 1.60, post-OP: 2.32, p = 0.009).

### 3.5 Correlation between preoperative symptom duration and postoperative pain intensity

Many patients exhibit chronic pain conditions. However, it remains unclear to what extent pudendal nerve decompression has a positive effect even after years of experiencing pain symptoms.

## 4 Pathophysiological alterations leading to PN

**Fig. 2:**
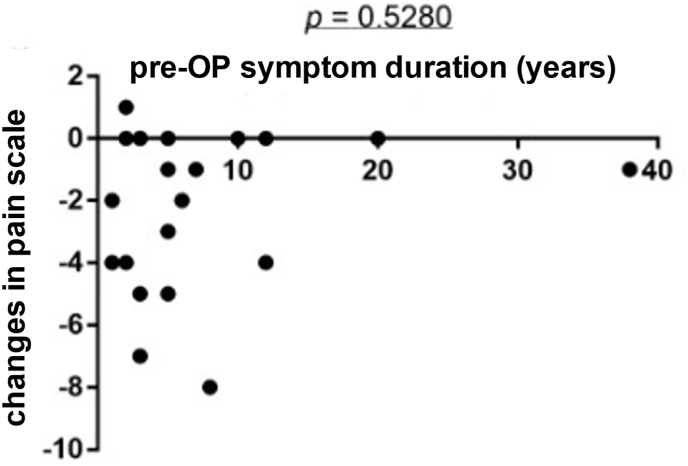
Correlation of preoperative pain duration and post-OP pain intensity. No significant correlation was found between pain duration and improvement in pain symptoms (p = 0.5280). Patients with a pain duration of less than ten years showed a greater decrease in pain intensity compared to those with a symptom duration of over ten years. While this trend was noticeable, it did not lead to significant results in the Pearson correlation analysis.

**Fig. 3:**
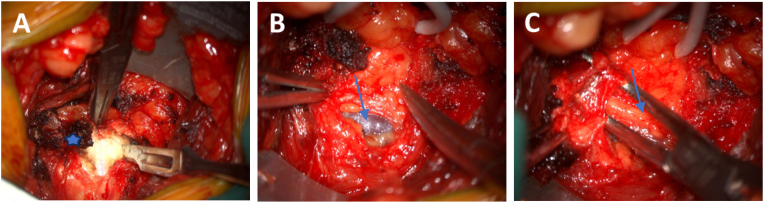
**Intraoperative pathophysiological changes leading to PN**. **A)** Right hypertrophic sacrotuberous ligament (asterisk), opened longitudinally, exhibiting an unusually thick and firm fiber structure covering the main trunk of the pudendal nerve and the inferior rectal nerve. **B)** an intra-extraneural varicose-altered vessel (arrow) **C)** Pudendal nerve (arrow) after transection of the sacrotuberous ligament and relief of the nerve.

In n = 15 cases (48%), a significant hypertrophy of the sacrotuberous ligament (see Fig. 2a) was identified, which exerted compression on the nerve. In n = 16 cases (52%) additional pathologies alongside ligament hypertrophy were identified. These included fibrous adhesions or attachments and a varicose intra-extraneural vessels (see Fig. 2b). Additionally, hypertrophy of the sacrospinous ligament was identified in n = 1 patient (see Fig. 3).

## 5 Discussion

PN remains one of the most controversial compression syndromes in peripheral nerve surgery. Work-up, diagnosis and patient's overall treatment is complex and demanding. Among a plethora of therapeutic options, surgical decompression plays a role in PN treatment. To better understand the impact of this surgical treatment our study was designed.

Pain perception in PN is a subjective parameter influenced by individual pain levels and various confounding factors in the treatment environment. However, surgery can be considered successful if the patient recognizes a personal benefit, primarily dependent on the relief of symptoms. Since there are no clinical signs for PN, pain perception is a key feature allowing the assessment of treatment success.

The limitations of this study are obvious: low sample size, retrospective design and the very subjective patient-reported questionnaire data.

In this study, the reduction in pain intensity served as the primary endpoint parameter. The postoperative follow-up period averaged 2.3 years (27 months), ranging from one year to a maximum of 5.3 years. Pain intensity measured on a visual analog scale (VAS) showed a statistically significant (p = 0.0002) mean reduction from an initial 7.9 points to 5.9 points after an average of 2.3 years. Of course, this is not a complete break-through in pain control, but as seen in the different aspects of daily life activities, patients QoL improved nonetheless.

Thus, a median decrease of 2 points in pain intensity was observed in 60% (n = 15/25) patients. N = 1 patient was pain free after decompression, n = 8 individuals experienced a substantial improvement of at least 50%, and another n = 6 showed a slight improvement in pain symptoms. N = 9 patients did not benefit from the surgery, and n = 1 participant reported an increase in pain by one point on the VAS.

Compared to other studies involving nerve decompression, a similar level of success was achieved. Popeney et al. (2007) observed a reduction in pain in 60% of n = 58 patients after twelve months, with n = 35 individuals experiencing at least a 50% relief, while 40% showed no improvement (Popeney et al., 2007). Robert et al. (2005) reported significant pain improvement in 71.4% of n = 16 patients in a surgical group compared to 13.3% in a non-surgical group after twelve months (Robert et al., 2005). Bautrant et al. (2003) found improvement in 86% of cases in a study with n = 104 patients (Bautrant et al., 2003). Beco et al. (2018) introduced a new technique, operative pudendoscopy, for decompressing the pudendal nerve using an endoscope through a transperineal approach (Beco et al., 2018). Twenty-four months post-operation, a significant improvement (p < 0.0001) in pain on the VAS was achieved, from 7.2 ± 1.4 points to 4.5 ± 2.9 points. Improvement was observed in 72% of patients, with a 50% pain reduction in 41.6%, while 16.8% did not benefit, and 10.6% showed deterioration from 6.6 ± 1.0 before the operation to 8.2 ± 1.0 after the operation.

The validated Marburg Questionnaire for Habitual Well-Being (Basler, 1999) was used to assess subjective well-being. Postoperatively, a significant improvement (p = 0.0001) in overall well-being was observed. N = 17 patients showed improvement, n = 4 reported unchanged overall well-being, and n = 4 indicated deterioration.

In a study by Popeney in 2007, following pudendal nerve decompression with a transgluteal approach and a 12-month follow-up, functional and QoL outcomes were examined in n = 58 patients (Popeney et al., 2007). The pre- and postoperative measurements were numerically assessed using the NIH-CPSI Impact Quality of Life questionnaire. A responder was defined as a patient experiencing a 50% or greater improvement in function and LQ, along with a minimum 50% reduction in pain on the VAS. 72% of patients reported preoperative limitations in performing everyday activities due to symptoms. Of these, 86% reported unhappiness or feeling bad when symptoms persisted. A comparison with the preoperative survey revealed an increased QoL in 60% of patients, with 40% not benefiting from the operation.

Similarly, Robert et al. compared in a randomized controlled study transgluteal decompression to non-surgical treatment in n = 16 patients each. After twelve months, the surgical group showed improvement in 71.4% compared to 13.3% in the non-surgical group, which received treatment with anticonvulsants, antidepressants, physiotherapy, and repeated pudendal nerve blocks (Robert et al., 2005).

Within this study, we adapted assessment of QoL on multiple levels of daily life allowing for a more precise picture of the impact of pudendal neuralgia and decompression therapy on subjective QoL. Following surgical decompression in our study, a significant improvement (p = 0.0001) in multidimensional well-being was achieved in n = 17 patients (68%). 20% were able to resume everyday activities, and there was a significant improvement (p = 0.0145) in participating in leisure activities for 44% of patients.

Overall, when considering all aspects of the questionnaire, postoperative improvement does not apply to the entire patient population. In n = 6 cases (24%), there was no positive change in all areas, indicating that these individuals did not benefit from surgical decompression.

To investigate whether preoperative symptom duration could serve as a predictor for the success of the operation, it was correlated using the Pearson Correlation Coefficient Test. The results did not yield a significant correlation (p = 0.5280) between the improvement in pain symptoms and the duration of chronic pain. This suggests that surgical decompression of the pudendal nerve is a viable option for patients with chronic conditions. These findings are in line with the findings of Mauillon et al., investigating the relationship between the postoperative result and the duration of symptoms, among other factors. The cohort consisted of n = 12 patients. N = 3 individuals were healed through decompression, n = 1 person experienced slight improvement, and n = 8 patients did not benefit from the operation. The duration of pain in non-healed patients was at least five years or longer in five cases; it was under five years in the three postoperatively pain-free patients (p = 0.15), indicating that preoperative symptom duration can be a predictor for the surgical outcome. However, the low number of study participants might be limiting (Mauillon et al., 1999). Robert et al. examined the outcomes of decompression following neurolysis and transposition of the pudendal nerve, resulting in a 67% reduction in pain for patients. They concluded that an early diagnosis of pudendal neuralgia might be a pivotal factor in enhancing postoperative pain relief and, consequently, influencing the overall success of the operation.

However, potentially other factors might have led to the non-respondence to pudendal decompression in this study. Regardless of careful medical history collection and findings, a contralateral inclusion in unilaterally operated patients could be causative for the lack of success. Moreover, due to sustained compression, irreversible nerve damage is conceivable. Perhaps its relief is not sufficient to achieve nerve regeneration and thus treat neuralgia.

Treatment-resistant chronic pain has significant emotional consequences and a psychological component, playing a crucial role in the pain process. Mauillon et al. investigated the relationship between depressive syndrome and the success of surgical decompression. In five out of six depressive patients, pudendal nerve neurolysis failed (Mauillon et al., 1999). Although no patients with severe depression are included in the study, such a psychological component could not be completely ruled out. One other potential source of error could be a misdiagnosis. Despite the informative clinical symptoms and a positive response to pudendal nerve blockade, another underlying pathomechanism resulting in chronic pain is possible.

Furthermore, incomplete nerve decompression must be considered. An inclusion in the distal Alcock's canal cannot be reached through the transgluteal approach, which was the surgical approach for most of our patients and thus, second look surgical approaches might be successful in pain relief in non-responding patients.

## 6 Conclusion

PN remains a difficult to treat entity. In our experience, decompression surgery may offer a promising intervention for patients with pudendal neuralgia who have not responded to conservative treatments, resulting in improvement in QoL in most of patients. However, further studies with larger patient numbers are required to corroborate our findings.

## Declaration of competing interest

The authors declare that they have no known competing financial interests or personal relationships that could have appeared to influence the work reported in this paper.
